# Deferred

**Published:** 1884-06

**Authors:** 


### DEFERRED.

We have marked for our pages a number of interesting extracts from that valuable journal, "Archives of Pediatrics," but they have so far been crowded out by the press of other matter. If any one wishes to see them before we can reproduce them, as well as to get much other information of value to those practicing dentistry, let him subscribe for the journal. It is now published in New York.

